# Supplementary material for: Dysregulation of mTOR signaling mediates common neurite and migration defects in both idiopathic and 16p11.2 deletion autism neural precursor cells
Source: eLife. 2024 Mar 25;13:e82809. doi: 10.7554/eLife.82809 (PMC11003747; doi:10.7554/eLife.82809)
Supplement: Figure 3—source data 1. — (A) Table showing the protein members in each enriched canonical pathway in I-ASD. (B) Table showing the protein members in each enriched canonical pathway in 16pDel ASD. [file elife-82809-fig3-data1.docx]

Figure 3-Figure supplement 1 A

| **Pathway** | **Proteins** | |
| --- | --- | --- |
| Tight Junction Signaling | AFDN,EPB41,MYH9,PTPA,STX4,TJP1,TJP2 | |
| mTOR Signaling | EIF3A,EIF4B,FKBP1A,MLST8,PTPA,RPS6,ULK1 | |
| Synaptogenesis Signaling Pathway | AFDN,ARHGEF7,CTNND1,FYN,MAP1B,MAPT,MARCKS | |
| Reelin Signaling in Neurons | AFDN,DCX,FYN,MAP1B,MAPT |  |
| Telomerase Signaling | HDAC2,HDAC4,HSP90AB1,PTGES3,PTPA | |
| HIPPO signaling | PPP1R7,PTPA,TJP2,YAP1 |  |
| Epithelial Adherens Junction Signaling | AFDN,BAIAP2,CTNND1,MYH9,TUBA1B | |
| AMPK Signaling | ARID1A,MLST8,PTPA,SMARCC1,ULK1 | |
| Phospholipase C Signaling | ARHGEF7,FYN,HDAC2,HDAC4,MARCKS | |
| Glucocorticoid Receptor Signaling | ARID1A,HMGB1,HSP90AB1,PTGES3,SMARCC1 | |

Figure 3-Figure supplement 1 B

| **Pathway** | **Proteins** |
| --- | --- |
| EIF2 Signaling | EIF3A,EIF3B,EIF4G1,EIF4G2,HNRNPA1,MAP2K2,PABPC1,PTBP1,RAF1,RPL15,RPL24,RPLP0,RPLP2,RPS10,RPS27A,RPS4X,RPS6,RRAS2 |
| p70S6K/EIF4 Signaling | EIF3A,EIF3B,EIF4EBP1,EIF4G1,EIF4G2,MAP2K2,PABPC1,PPP2R5D,RAF1,RPS10,RPS27A,RPS4X,RPS6,RRAS2 |
| mTOR Signaling | ATG13,EIF3A,EIF3B,EIF4B,EIF4EBP1,EIF4G1,EIF4G2,MLST8,PPP2R5D,PRKCA,RPS10,RPS27A,RPS4X,RPS6,RRAS2 |
| Spliceosomal Cycle | RBMX2,SF3A1,SF3B1,SF3B2,SF3B3,SNRNP200,U2AF2 |
| ERK/MAPK Signaling | BCAR1,EIF4EBP1,FYN,HSPB1,MAP2K2,PAK2,PPP1R12A,PPP1R7,PPP2R5D,PRKCA,RAF1,RRAS2,YWHAQ |
| Cell Cycle Control | CDK11B,CDK12,CDK13,LIG1,MCM2,MCM3,TOP2A |
| NER Pathway | CHAF1A,DDB2,HMGN1,LIG1,POLD3,POLR2A,TCEA1,TOP2A,XPC |
| Telomerase Signaling | HDAC4,HSP90AA1,HSP90AB1,MAP2K2,PPP2R5D,PTGES3,RAF1,RRAS2,SP1 |
| HIPPO signaling | AJUBA,PPP1R12A,PPP1R7,PPP2R5D,TJP2,TP53BP2,YAP1,YWHAQ |
| Actin Cytoskeleton Signaling | ABI2,BAIAP2,BCAR1,CFL1,FLNA,MAP2K2,MYH9,PAK2,PPP1R12A,RAF1,RRAS2,VCL |

Figure 3-Figure supplement 5 B

| **Pathway** | **Proteins** |
| --- | --- |
| O-linked glycosylation | GALNT3,LARGE2,MUC12,MUC16,MUC17,MUC19,MUC2,MUC21,  MUC3A,MUC4,MUC5AC,MUC6,MUC7,ST3GAL3 |
| MHC Class 1 Signaling | ICAM3, CCR7,FSCN2,HLA-C,HLA-DRB1,HLA-DRB5,HLA-G,ICAM3,TLN1, LATS1, TGF1B, CBLB |
| Interleukin signaling | CSF1,CSF1R,IFNLR1,TYK2 |
| Antigen Presentation Pathway | HLA-C,HLA-DQB1,HLA-DRB1,HLA-DRB5,HLA-G |
| Generic Transcription Pathway | AR,HDAC7,MED14,RORB,ZKSCAN8,ZNF285,ZNF33A,ZNF417/ZNF587,  ZNF439,ZNF492/ZNF98,ZNF493,ZNF506,ZNF554,ZNF560,  ZNF567,ZNF678,ZNF717,ZNF726,ZNF729,ZNF750,ZNF782 |
| NAD Phosphorylation and Dephosphorylation | ACP3,ACP5,ACP6 |
| Peroxisomal Protein Regulation | ACOX1,SCP2, ACOX1,HAO1,MLYCD,PAOX,SCP2 |
| Chaperone Mediated Autophagy | CFTR,GFAP,PCNT |
| Activation of gene expression by SREBF (SREBP) | ACACB,FASN,LSS,TGS1 |
| Regulation of the Epithelial Mesenchymal Transition in Development Pathway | APC,BCL9,CDH1,GLI2,PSEN1,PTCH2 |
| Lanosterol Biosynthesis | LSS |
| Endocannabinoid Neuronal Synapse Pathway | CACNA1H,CACNA2D4,CACNG1,CNR1,DNAH10,GNB1L,MAPK6,PLCE1 |
