# Supplementary material for: Dysregulation of mTOR signaling mediates common neurite and migration defects in both idiopathic and 16p11.2 deletion autism neural precursor cells
Source: eLife. 2024 Mar 25;13:e82809. doi: 10.7554/eLife.82809 (PMC11003747; doi:10.7554/eLife.82809)
Supplement: Supplementary file 1. — In each cell, multiple different kinds of N-values are represented: # of clones (C)/Total # of neural inductions (NI)/# of experiments (E) and for neurite experiments, # of dishes (D) whereas for neurospheres experiment, # of neurospheres (NS). [file elife-82809-supp1.docx]

**Supplemental Table 1**

| **Figure** | **Subfigure** | | |
| --- | --- | --- | --- |
|  | **B (Neurites)** | **B (Neurites)** | **B (Neurites)** |
| **1** | Sib-1  N= 5C/11 NI/88E/200D | SIB-2  n=3C/6NI/34E/100D | SIB-3  n=2C/6NI/15E/45E |
|  | I-ASD-1  N=5C/11 NI/70E/210D | I-ASD-2  n=3C/6NI/32E/75E | I-ASD-3  n=2C/6NI/12E/40E |
|  | **D (Neurospheres, NS)** | **D (Neurospheres, NS)** | **D (Neurospheres, NS)** |
|  | Sib-1  N= 3C/3NI/13E/212NS | SIB-2  n=3C/3NI/5E/110NS | SIB-3  n=2C/2NI/4E/84NS |
|  | I-ASD-1  N=4C/4NI/11E/170NS | I-ASD-2  n=3C/3NI/4E/80NS | I-ASD-3  n=2C/2NI/3E/63NS |
|  | **E (Neurites)** | **F (Neurospheres, NS)** |  |
|  | All Sibs: See 1B  All I-ASD: See 1B | All Sibs: See 1B  All I-ASD: See 1B |  |
|  | NIH-1: 1C/2NI/9E/27D  NIH-2: 1C/2NI/11E/33D  NIH-3: 1C/2NI/9E/27D | NIH-1: 1C/2NI/3E/40NS  NIH-2: 1C/2NI/2E/35NS  NIH-3: 1C/2NI/2E/20NS |  |
|  | 16pDel-M-1: 2C/3NI/16E/42D  16pDel-M-2: 2C/3NI/19E/48D  16pDel-F: 2C/3NI/11E/30D | 16pDel-M-1: 2C/3NI/4E/50NS  16pDel-M-2: 2C/3NI/4E/48NS  16pDel-F: 2C/3NI/3E/52NS |  |
| **Figure** | **Subfigure** | | |
|  | **A (Neurites)** | **B (Neurites)** | **C (Neurites)** |
| **2** | Sib-1: 5C/8NI/24E/55D  Sib-2: 3C/5NI/10E/25D  Sib-3: 2C/6NI/6E/18D | Sib-1: 3C/6NI/15E/34D  Sib-2: 3C/4NI/12E/25D  Sib-3: 2C/4NI/6E/18D | All Sibs: See 2A  NIH-1: 1C/2NI/3E/7D  NIH-2: 1C/2NI/3E/7D  NIH-3: 1C/2NI/3E/7D |
|  | I-ASD-1: 5C/8NI/20E/50D  I-ASD-2: 3C/5NI/12E/25D  I-ASD-3: 2C/6NI/6E/18D | I-ASD-1: 3C/6NI/16E/35D  I-ASD-2: 3C/4NI/13E/26D  I-ASD-3: 2C/4NI/6E/18D | All I-ASD: See 2A  16pDel-M-1: 2C/2NI/7E/16D  16pDel-M-2: 2C/2NI/7E/18D  16pDel-F: 2C/2NI/5E/13D |
|  | **D (Neurites)** | **E (Neurospheres, NS)** | **F (Neurospheres, NS)** |
|  | All Sibs: See 2A  NIH-1: 1C/2NI/3E/7D  NIH-2: 1C/2NI/3E/7D  NIH-3: 1C/2NI/3E/7D | Sib-1: 3C/7NI/9E/175NS  Sib-2: 3C/4NI/5E/90NS  Sib-3: 2C/3NI/4E/84NS | All Sib: See 2E  NIH-1: 1C/2NI/3E/42 NS  NIH-2: 1C/2NI/3E/38 NS  NIH-3: 1C/2NI/2E/30 NS |
|  | 16pDel-M-1: 2C/3NI/7E/16D  16pDel-M-2: 2C/3NI/7E/15D  16pDel-F: 2C/3NI/5E/13D | I-ASD-1: 3C/6NI/9E/170NS  I-ASD-2: 3C/3NI/4E/60NS  I-ASD-3: 2C/3NI/3E/63NS | All Sibs: See 2E  16pDel-M-1: 2C/2NI/4E/55NS  16pDel-M-2: 2C/2NI/3E/34NS  16pDel-F: 2C/2NI/3E/38NS |
| **Figure** | **Subfigure** | | |
|  | **B (Westerns)** | **D (Westerns)** | **E (Westerns)** |
| **4** | Sib-1: 3C/5NI/5E  I-ASD-1: 4C/6NI/6E | Sib-3: 2C/4NI/3E  I-ASD-3: 4C/3NI/3E | Sib-2: 3C/5NI/4E  I-ASD-2: 3C/5NI/4E |
|  | **H** | **H** |  |
|  | All Sibs: Expts: 9, see above  NIH-1: 1C/2NI/3E  NIH-2: 1C/2NI/3E  NIH-3: 1C/2NI/3E | 16pDel-M-1: 2C/2NI/3E  16pDel M-2: 2C/2NI/3E  16pDel-F: 2/C/2NI/3E |  |
| **Figure** | **Subfigure** | | |
|  | **B (Westerns)** | **C (Neurites)** | **D (Neurospheres, NS)** |
| **5** | Sib-1: 3C/5NI/3E  I-ASD-1: 3C/5NI/3E | Sib-1: 3C/5NI/5E/13D  I-ASD-1: 3C/5NI/5E/13D | Sib-1: 2C/3NI/3E/60NS  I-ASD-1: 2C/3NI/60NS |
|  | **F (Westerns)** | **G (Neurites)** | **H (Neurospheres, NS)** |
|  | Sib-1: 3C/3NI/3E  I-ASD-1: 3C/3NI/3E | Sib-1: 3C/5NI/5E/13D  I-ASD-1: 3C/5NI/5E/13D | Sib-1: 2C/3NI/3E/60NS  I-ASD-1: 2C/3NI/60NS |
|  | **I (Neurites)** | **J (Neurites)** |  |
|  | Sib-3: 2C/3NI/3E/9D  I-ASD-3: 2C/3NI/3E/9D | Sib-3: 2C/3NI/3E/9D  I-ASD-3: 2C/3NI/3E/9D |  |
| **Figure** | **Subfigure** | | |
|  | **B (Westerns)** | **C (Neurites)** | **D (Neurospheres, NS)** |
| **6** | Sib-2: 3C/4NI/3E  I-ASD-2: 3C/4NI/3E | Sib-2: 3C/4NI/4E/12D  I-ASD-2: 3C/4NI/4E/12D | Sib-2: 3C/3NI/3E/60NS  I-ASD-2:3C/3NI/3E/60NS |
|  | **F (Westerns)** | **G (Neurites)** | **H (Neurospheres, NS)** |
|  | Sib-2: 3C/4NI/3E  I-ASD-2: 3C/4NI/3E | Sib-2: 2C/3NI/3E/9D  I-ASD-2: 2C/3NI/3E/9D | Sib-2: 2C/2NI/2E/42NS  I-ASD-2: 2C/2NI/2E/42NS |
|  | **I (Neurites)** | **J (Neurites)** |  |
|  | NIH-1: 1C/2NI/3E/8D  NIH-2: 1C/2NI/3E/8D | NIH-1: 1C/2NI/3E/8D  NIH-2: 1C/2NI/3E/8D |  |
|  | 16pDel-M-1: 2C/3NI/3E/8D  16pDel-M-2: 2C/3NI/3E/8D | 16pDel-M-1: 2C/3NI/3E/8D  16pDel-M-2: 2C/3NI/3E/8D |  |
| **Figure** | **Subfigure** | | |
|  | **A (Neurites)** | **B (Neurites)** | **C (Neurites)** |
| **7** | I-ASD-1: 2C/3NI/3E/9D | Sib-1: 2C/3NI/3ED/9D | Sib-1: 2C/3NI/3ED/9D |
|  | **D (Neurites)** |  |  |
|  | I-ASD-2: 2C/3NI/3E/8D |  |  |
