# Supplementary material for: Dysregulation of mTOR signaling mediates common neurite and migration defects in both idiopathic and 16p11.2 deletion autism neural precursor cells
Source: eLife. 2024 Mar 25;13:e82809. doi: 10.7554/eLife.82809 (PMC11003747; doi:10.7554/eLife.82809)
Supplement: Supplementary file 2. — The count of the heterozygous and homozygous alternative allele genotypes and their ratio in the chr16.p11.2 deletion region (chr16: 28,500,001–35,300,000). In the event of a chr16.p11.2 deletion, we expect no heterozygous genotypes in the region. The large number of heterozygous genotypes across all individuals in this region indicates that the deletion does not appear in any of the individuals in the three I-ASD families. [file elife-82809-supp2.docx]

**Supplemental Table 2:**

| **Sequenced Individual** | **# of heterozygous alternative genotypes** | **# of homozygous alternative genotypes** | **% of alternative heterozygous genotypes** |
| --- | --- | --- | --- |
| Family-1 Male Parent SL128062 | 15889 | 20041 | 44.2% |
| Family-1 Female Parent SL128065 | 12890 | 23040 | 35.9% |
| Family-1 LLI  07C69064 | 11875 | 24055 | 33.1% |
| I-ASD-1  2743-SL-0029 | 9224 | 26706 | 25.7% |
| Sib-1  07C69065 | 15305 | 20625 | 42.6% |
| Family-2 Male Parent SL128102 | 17859 | 18071 | 49.7% |
| Family-2 Female Parent SL128091 | 14600 | 21330 | 40.6% |
| I-ASD-2  07C65371 | 15688 | 20242 | 43.7% |
| Sib-2  07C66696 | 15536 | 20394 | 43.2% |
| Family-3 Male Parent SL127748 | 16822 | 19108 | 46.8% |
| Family-3 Female Parent  SL127734 | 14769 | 21161 | 41.1% |
| Family-3 LLI SL127742 | 16785 | 19145 | 46. 7% |
| Sib-F  05C40413 | 14687 | 21243 | 40.9% |
| Sib-3  05C40411 | 15140 | 20790 | 42.1% |
| I-ASD-3  2743-SL-0028 | 11341 | 24589 | 31.6% |
